# Experimental data on compressive strength and durability of sulfur concrete modified by styrene and bitumen

**DOI:** 10.1016/j.dib.2017.05.030

**Published:** 2017-05-19

**Authors:** M. Dehestani, E. Teimortashlu, M. Molaei, M. Ghomian, S. Firoozi, S. Aghili

**Affiliations:** Faculty of Civil Engineering, Babol Noshirvani University of Technology, Babol, Iran

**Keywords:** Sulfur concrete, Styrene, Bitumen, Ignition, Acid resistance, Compressive strength

## Abstract

In this data article experimental data on the compressive strength, and the durability of styrene and bitumen modified sulfur concrete against acidic water and ignition are presented. The percent of the sulfur cement and the gradation of the aggregates used are according to the ACI 548.2R-93 and ASTM 3515 respectively. For the styrene modified sulfur concrete different percentages of styrene are used. Also for the bitumen modified sulfur concrete, different percentages of bitumen and the emulsifying agent (triton X-100) are utilized. From each batch three 10×10×10 cm cubic samples were casted. One of the samples was used for the compressive strength on the second day of casting, and one on the twenty-eighth day. Then the two samples were put under the high pressure flame of the burning liquid gas for thirty seconds and their ignition resistances were observed. The third sample was put into the acidic water and after twenty eight days immersion in water was dried in the ambient temperature. After drying its compressive strength has been evaluated.

## **Specifications Table**

**Table:** 

Subject area	*Civil Engineering*
More specific subject area	*Concrete Technology, Sulfur Concrete*
Type of data	*Table, Figure*
How data was acquired	*Casting concrete samples in the laboratory*
Data format	*Raw*
Experimental factors	*The sulfur concrete was studied in the literature*[1], [2], [3]*and some samples of pure sulfur concrete were casted that the data wasn׳t recorded.*
Experimental features	*Using styrene and bitumen as modifiers of sulfur concrete*
Data source location	*Noushirvani Institute of Technology, Babol, Iran*
Data accessibility	*Data are presented in this article*

## V**alue of the data**

• The data present the properties of concrete for different percentages of the modifiers.
• The data provide a useful point that by changing the percentages of the sulfur, the modifiers, and the emulsifying agent can reach to an optimum result.
• The data could be helpful for achieving the other properties of these kinds of modified sulfur concretes.

## 1 Data

The data include information on the properties of modified sulfur concrete:

– The properties of samples with different percentages of modifiers and different periods of chemical reaction time (different stirring times)
– Compressive strength on the second and twenty-eighth day of casting
– Compressive strength of a sample after twenty eight days immersion in acidic water
– Ignition resistance of the samples after thirty seconds being under a high pressure flame of burning liquid gas

## 2 Experimental design, materials and methods

### 2.1 Experimental design

**Fig. 1:**
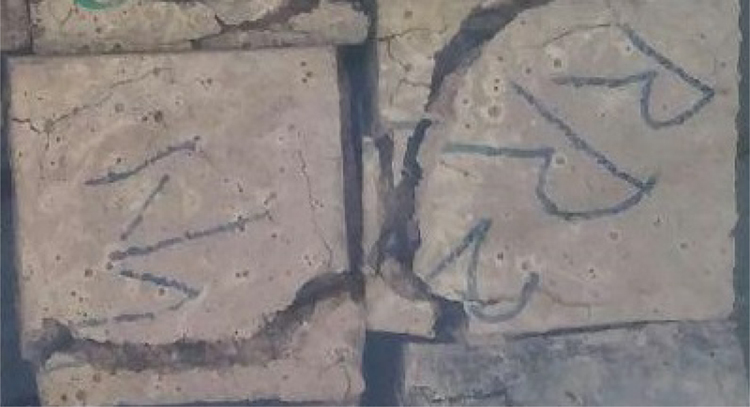
Styrene modified concrete samples in water labeled ׳Failed׳.

**Fig. 2:**
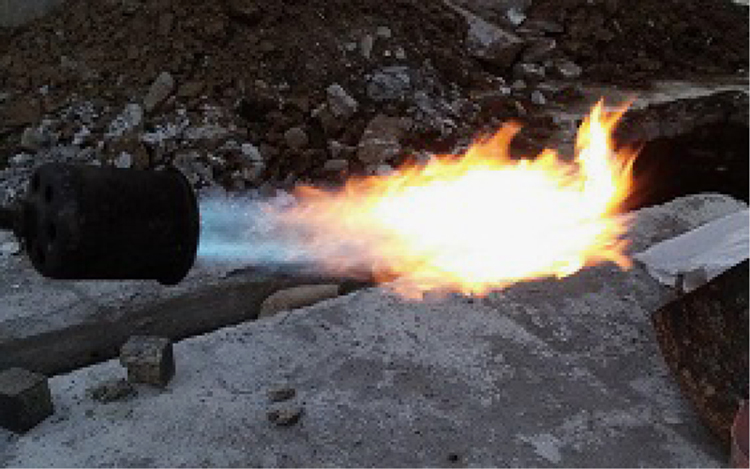
High pressure flame of the burning liquid gas.

**Fig. 3:**
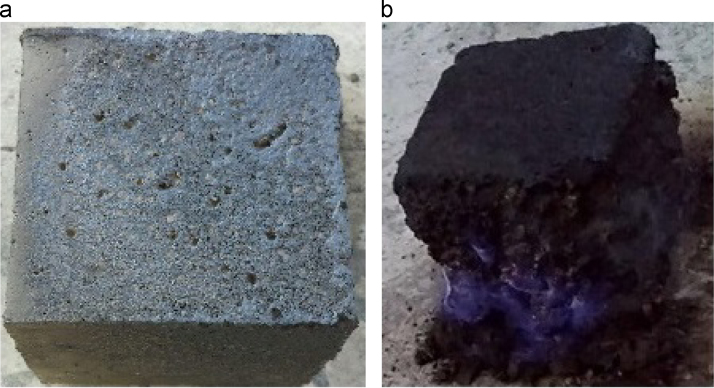
Ignition resistance of the concrete samples, (a) not dangerous labeled (b) dangerous labeled.

**Fig. 4:**
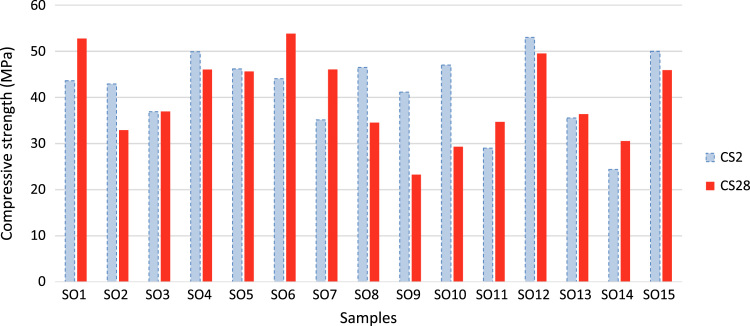
Compressive strengths of the series 1 of the styrene modified concrete samples on the day 2 (CS2) and 28 (CS28).

**Fig. 5:**
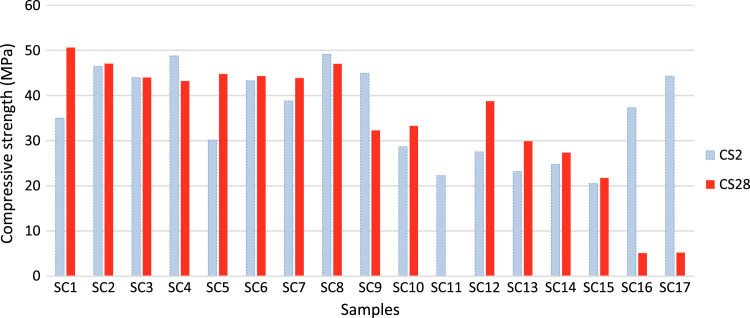
Compressive strengths of the series 2 of the styrene modified concrete samples on the day 2 (CS2) and 28 (CS28).

**Fig. 6:**
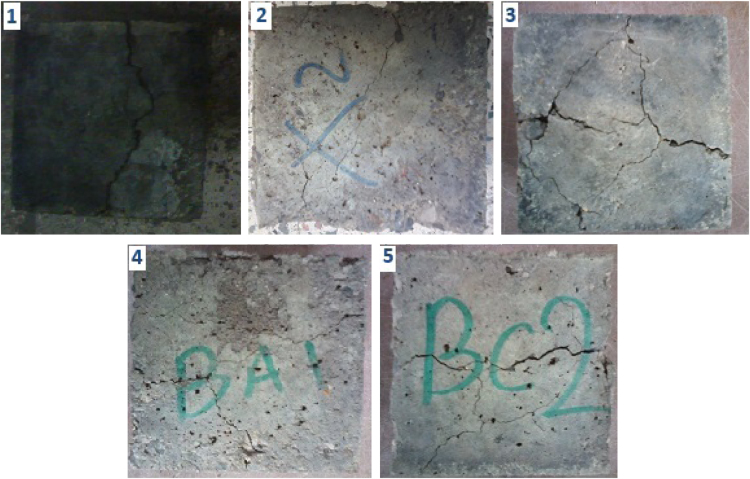
The bitumen modified concrete samples after 28 days immersion in acidic water, (1) B3, (2) B2, (3) B5, (4) B6, and (5) B7.

**Fig. 7:**
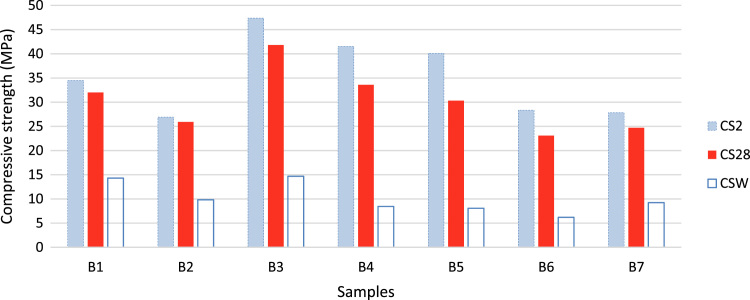
Compressive strengths of the bitumen modified concrete samples on the day 2 (CS2) and 28 (CS28), and after 28 days immersion in acidic water (CSW).

The data include three series of samples, two series of styrene modified samples ( Table 1, Table 2, Table 3, and Fig. 4, Fig. 5), and one series of bitumen modified samples (Table 4 and Fig. 7). For all series constant mixture proportions are used. According to ACI 548.2R-93 for the maximum aggregate size of 0.5 in (12.7 mm) the percent by weight of the cement (sulfur and the modifier) has been used as 17%. From each batch three 10×10×10 cm cubic samples were casted. One of the samples was used for the compressive strength on the second day of casting (CS2 in the figures), and one on the twenty-eighth day (CS28 in the figures). In addition the CS28 for the sample SC11 was not taken (Fig. 5). After taking the compressive strength the two samples were put under the high pressure flame of the burning liquid gas (Fig. 2) for thirty seconds and their ignition resistances were seen. In the tables the symbol ׳IgRn׳ represents the sample׳s ignition resistance on the day ׳n׳ (2 & 28) of casting. The ignition of some of the samples was progressive and the sample would crumble, these samples are labeled ׳dangerous׳ (Fig. 3). On the other hand the ignition of some of the samples would be stopped after some minutes, these samples are labeled ׳not dangerous׳ (Fig. 3). It should be noted that for some of the samples it was not possible to check the ignition resistance. For the samples in the Table 3, Table 4 the ignition resistance on the day 28 was not taken. The third sample was put into the acidic water and after twenty eight days immersion in water it was dried in the ambient temperature, so after drying it׳s compressive strength was taken. The symbol ׳CSW׳ represents the sample׳s compressive strength after 28 days immersion in acidic water. Some of the styrene modified samples after few hours or few days being in water, had excessive cracks that it was not possible to take their compressive strength, these samples are labeled ׳failed׳ (Fig. 1). In Table 3 the CSW is not shown and all samples were failed. Also the cracks of bitumen modified samples after immersion in acidic water are shown in Fig. 6. Although having some cracks it was possible to take the compressive strength for these samples (Fig. 7).

**Table 1:** Aggregate gradation according to ASTM 3515.

Sieve size	3/4 in	1/2 in	No. 4	No. 8	No. 50	No. 200
Percent passing	100	90	62	44	15	8

**Table 2:** Series 1 of the styrene modified concrete samples׳ properties (with the open opening of the pot).

Sample	Styrene percent (%)	Stirring time (h)	CSW (MPa)	IgR2	IgR28
SO1	2	0.5	18.16	Not dangerous	Dangerous
SO2	2	1	failed	Not dangerous	Dangerous
SO3	2	1.5	failed	Not dangerous	Dangerous
SO4	2	2	31.39	Not dangerous	Not dangerous
SO5	2	3	24.57	Not dangerous	Dangerous
SO6	4	0.5	21.08	Dangerous	Dangerous
SO7	4	1.5	failed	Not dangerous	Not dangerous
SO8	4	2	failed	Not dangerous	Dangerous
SO9	10	3	failed	Dangerous	Dangerous
SO10	15	3	failed	Wasn׳t done	Dangerous
SO11	20	2	failed	Not dangerous	Not dangerous
SO12	20	4	failed	Not dangerous	Dangerous
SO13	25	2	failed	Wasn׳t done	Wasn׳t done
SO14	25	3	failed	Wasn׳t done	Dangerous
SO15	25	4	failed	Not dangerous	Not dangerous

**Table 3:** Series 2 of the styrene modified concrete samples׳ properties (with the closed opening of the pot).

Sample	Styrene percent (%)	Stirring time (h)	IgR2
SC1	2	0.5	Not dangerous
SC2	2	1	Not dangerous
SC3	2	1.5	Not dangerous
SC4	2	2	Dangerous
SC5	4	0.5	Not dangerous
SC6	4	1	Dangerous
SC7	4	1.5	Dangerous
SC8	4	2	Dangerous
SC9	5	6	Not dangerous
SC10	7.5	6	Dangerous
SC11	10	4	Dangerous
SC12	10	6	Not dangerous
SC13	15	4	Wasn׳t done
SC14	15	6	Dangerous
SC15	20	4	Wasn׳t done
SC16	25	4	Wasn׳t done
SC17	30	4	Dangerous

**Table 4:** Bitumen modified concrete samples׳ properties.

Sample	Bitumen percent (%)	Stirring time (h)	Triton x-100 (cc)	IgR2
B1	2.5	2	0	Not dangerous
B2	2.5	1	0.4	Not dangerous
B3	1	1	0.4	Not dangerous
B4	2	1	0.6	Wasn׳t done
B5	2.5	3	0.7	Wasn׳t done
B6	3	1	0.9	Wasn׳t done
B7	3	3	0.9	Wasn׳t done

### 2.2 Materials

The sulfur concrete consists of the sulfur cement and the aggregates. The sulfur cement comprises of the sulfur and the modifier. So the modifiers are styrene and bitumen. The granular sulfur (99.9%) was bought from Tehran refinery, Iran. The styrene had a boiling point of 145 °C, and a specific gravity at 20 °C of 0.909 g/cm^3^. The bitumen was a 60/70 one with a softening point of 48.7 °C, specific gravity at 20 °C of 1.0287 g/cm^3^, kinematics viscosity at 135 °C of 429 cSt. The aggregates were natural crushed stones and the gradation of the aggregates was according to the ASTM 3515 (Table 1). Also for the bitumen modified sulfur cement an emulsifying agent named triton X-100 (iso-octylphenoxy polyethoxy ethanol) was used.

### 2.3 Methods

**Fig. 8:**
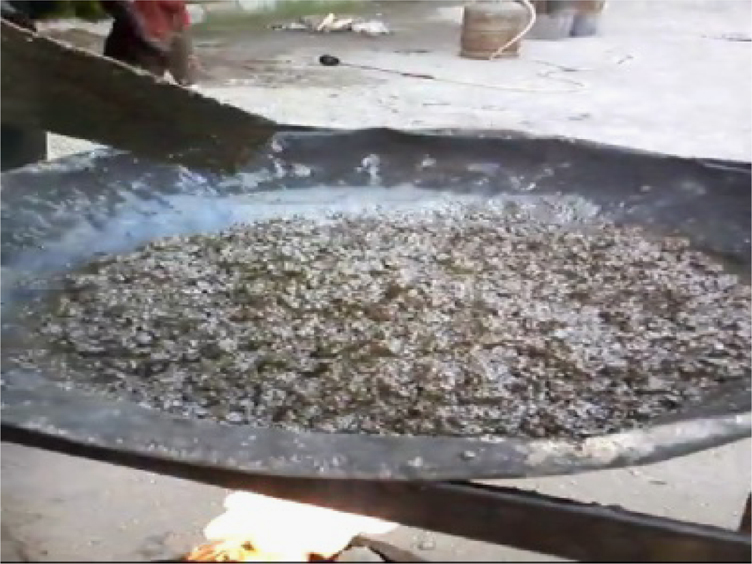
Blending the sulfur cement and the heated aggregates and achieving the sulfur concrete.

**Fig. 9:**
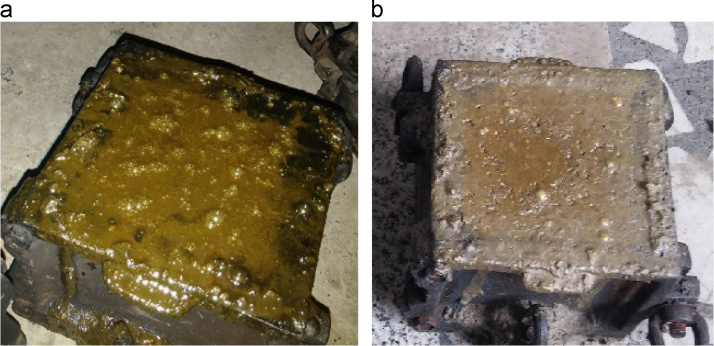
(a) Fresh concrete (b) Casted concrete after few hours.

To obtain the sulfur cement the elemental sulfur and different percentages of the modifiers and the emulsifying agent (for the bitumen modified cement) have been mixed in a pot, and the pot was in an oil bath. So then the mixture was heated to 137–140 °C and stirred for a period of time. Finishing the period the sulfur cement was achieved. Simultaneously the aggregates were heated to 130 °C in an oven. Afterward the filler was added to the cement and then were mixed, next the mixture was added to the aggregates and got blended thoroughly (Fig. 8). The product was concrete that was casted in the molds that were heated to 130 °C (Fig. 9). Also the vibration was done on a vibration table. It should be noted that for the styrene modified concrete at first the sulfur was heated to 135 °C and then the styrene was added. Besides for the bitumen modified concrete the bitumen was added to the sulfur at first and the emulsifying agent (triton X-100) was added at 135 °C.

**Fig. 10:**
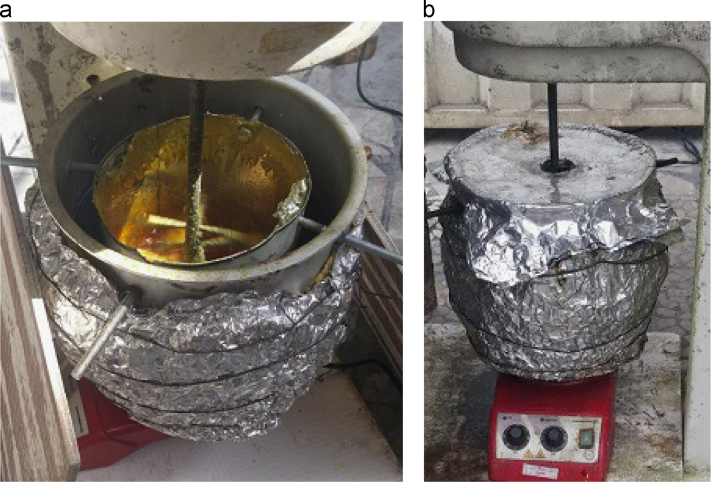
The mixing of the sulfur and the modifier in two situations (a) series 1, with the open opening of the pot (b) series 2, with the closed opening of the pot.

The mixing of the sulfur and the modifier was done in two situations. For one series of the styrene modified concrete samples (series 1) the opening of the pot was open and the vapors would exit (Table 2, and Fig. 10a). But for the other series of the styrene modified (series 2) and also for the bitumen modified samples the opening of the pot was closed and just an opening was open for the stirring rod to path through (Tables 3 and 4, Fig. 10b).

The modifiers were used with different proportions of the weight of cement (sulfur plus modifier). Also different volumes of the triron X-100 in proportion with different quantities of bitumen were utilized in the bitumen modified concrete.
