# Supplementary material for: Experimental data on compressive strength and durability of sulfur concrete modified by styrene and bitumen
Source: Data Brief. 2017 May 19;13:137–44. doi: 10.1016/j.dib.2017.05.030 (PMC5451180; doi:10.1016/j.dib.2017.05.030)
Supplement: Supplementary file 1 — Supplementary material [file mmc1.docx]

All authors disclose no financial and personal relationships exist with other people or organizations that could inappropriately influence (bias) their work. There are no conflicts of interest.

Conflicts of interest: none
